# Ectopic pancreas in the anterior mediastinum: A report of two cases and review of the literature

**DOI:** 10.3892/ol.2014.1840

**Published:** 2014-01-29

**Authors:** LIZHI ZHANG, LI-QING PENG, JIAN-QUN YU, HONG-MEI YUAN, ZHI-GANG CHU, HAN-JIANG ZENG, BING WEI

**Affiliations:** 1 Department of Radiology, West China Hospital, Sichuan University, Chengdu, Sichuan 610041, P.R. China; 2 Department of Pathology, West China Hospital, Sichuan University, Chengdu, Sichuan 610041, P.R. China

**Keywords:** ectopic pancreas, computed tomography, magnetic resonance imaging, anterior mediastinum

## Abstract

Ectopia of the pancreatic tissue is a developmental anomaly found in ~2% of all autopsies, and 70~90% of these anomalies are located in the gastrointestinal tract. Mediastinal localization of an ectopic pancreas is extremely rare. Herein, we report two cases with mediastinal ectopic pancreas clarified by pathology and shown by thoracic contrast-enhanced computed tomography (CT) and magnetic resonance imaging (MRI). In addition, a brief review of the relevant literatures is presented. Although CT and MRI manifestations of this lesion are nonspecific, certain notable findings need to be focused on. When there is a mass in the anterior mediastinum with marked and heterogeneous enhancement, along with necrotic and liquefied non-enhanced areas in the center, ectopic pancreas should be considered and differentiated from other neoplasms in this region.

## Introduction

An ectopic pancreas is pancreatic tissue which lacks anatomic and vascular continuity with the main body of the pancreas, it is also known as an aberrant pancreas or heterotopia of the pancreas (1–12). Although there are two different hypotheses, the etiology of ectopic pancreas remains unclear (10). An ectopic pancreas is a developmental anomaly normally located in the gastrointestinal tract, including the stomach, duodenum, jejunum and ileum (7,8). An ectopic pancreas in the mediastinum is particularly uncommon. To the best of our knowledge, since the existence of anterior mediastinal ectopia of pancreatic tissue was verified pathologically by Klob in 1859 for the first time (1), there are only 12 cases in the English literature up to January 2013 (2–12). Herein, we present the clinical, radiological and histopathological findings of an uncommon ectopic pancreas in the anterior mediastinum for a better understanding of this entity.

## Case reports

### Case 1

**Figure 1:**
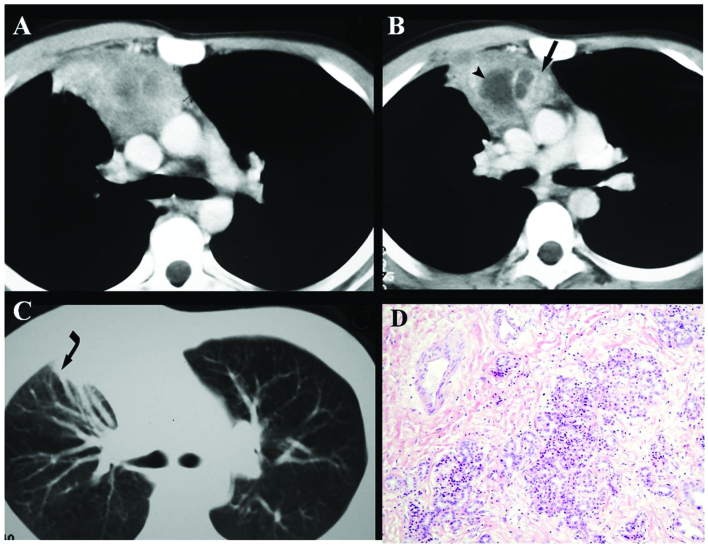
Contrast-enhanced computed tomography (CT) in case 1 revealed (A) an irregular soft tissue mass in the anterior mediastinum with (B) marked and heterogeneous enhancement (straight arrow), with the exception of the necrotic and liquefied non-enhanced area in the center (arrow head). (C) The mass invaded the anterior segment of the upper lobe of the right lung (curved arrow). (D) Microscopic images revealed abnormally differentiated and disorganized pancreatic lobules with acini, ducts, islets and malformed vessels. The pancreatic lobular architectures of the lesion are sparser than that of the normal pancreatic tissue (magnification, ×200; hematoxylin-eosin stain).

A 15-year-old male was referred to West China Hospital, Sichuan University (Chengdu, China) with complaints of chest pain, coughing and fever. There were no positive findings on physical examination and laboratory studies. A contrast-enhanced computed tomography (CT) scan was subsequently performed on the thorax. As shown on enhanced CT images, an irregular soft tissue mass measuring 7.0×4.5 cm was identified in the anterior mediastinum and extended to the anterior segment of the right upper lung, with partial consolidation. The mass was markedly and heterogeneously enhanced, whereas necrotic and liquefied non-enhanced areas were observed in the center of the mass. The lesion obscured the anterior chest wall and adjacent vascular structures, including the ascending aorta and superior vena cava (Fig. 1A–C). No destruction of the sternum and ribs, and no pleural effusion were found. A provisional diagnosis of thymoma was suspected.

The chest was opened by a median sternotomy incision. A solid and hard mass in the anterior mediastinum had firm adhesions to the superior vena cava, left brachiocephalic vein, pericardium, the mediastinal pleura and anterior chest wall. The anterior segment of the right upper lung was also invaded. The mass and part of the anterior segment of the right upper lung were together removed. Since no malignancy was reported from the frozen-section of the lesion, no further dissection was performed.

Histopathologically, the lesion exhibited abnormally differentiated and disorganized pancreatic lobules with acini, ducts, islets and malformed vessels. The pancreatic lobular architectures of the lesion were sparser than that of the pancreatic tissue located in the normal anatomic site (Fig. 1D). Therefore, an ectopic pancreas with encapsulated fibrous tissue without clear boundary in the anterior mediastinum was confirmed. Following surgery, the patient remained well and was asymptomatic with no evidence of recurrence or metastasis during a follow-up period of eight years.

### Case 2

A 16-year-old female with a six-month history of throat discomfort and neck swelling was referred to West China Hospital for further evaluation. Physical examination revealed that the soft tissue mass arising from the abdomen was soft, and the patient experienced tenderness on deep palpation. Routine laboratory findings were within normal range.

**Figure 2:**
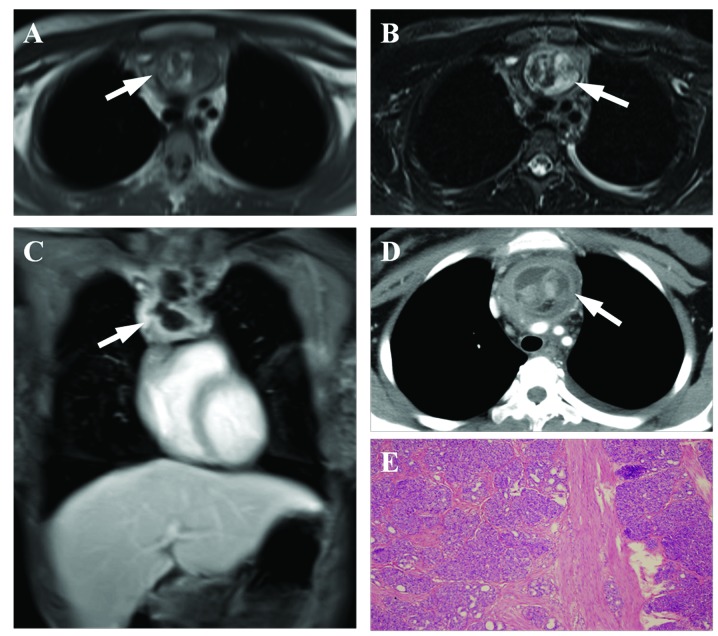
A 16-year-old female (case 2) with ectopic pancreas in the anterior mediastinum. (A) T1-weighted image and (B) T2-weighted image with fat suppression indicating a heterogeneous lesion (white arrow) containing fat and necrotic components. (C) Contrast-enhanced coronal T1-weighted and (D) axial computed tomography images show marked and heterogeneous enhancement with the exception of the necrotic area. (E) Histologically, ectopic pancreatic tissues were detected (magnification, ×80; hematoxylin-eosin stain).

Chest radiograph demonstrated a soft tissue mass in the anterior mediastinum. Unenhanced CT findings revealed a 6-cm heterogeneous mass at the region of the anterior mediastinum. Within the mass, there was an area of adipose tissue. There was no calcification in the mass. The mass enhanced slightly following intravenous administration of contrast material. MRI showed a heterogeneous anterior mediastinum mass with fat, solid and cystic components (Fig. 2A–D). The thymus itself was not identified. The poorly circumscribed mass invaded the adjacent structures, including the pericardium, mediastinal vessels, anterior tracheal wall and the lower pole of the thyroid. A small amount of pleural effusion was found. No sign of bony involvement was revealed. A tentative pre-operative diagnosis of a malignant tumor of undetermined origin, teratoma or invasive thymoma was considered on the basis of the imaging findings. Surgical resection was then performed under anesthesia. A mass located posterior to the sternum was found, which adhered to the superior vena cava, innominate vein, the adjacent pericardium and pleura. There was no evidence of abnormal lymph nodes or lung involvement.

Histologically, the mass was finally diagnosed as an ectopic pancreas in the mediastinum (Fig. 2E). Postoperatively, the patient recovered uneventfully and no recurrence or metastasis was observed during the six months of follow-up.

This study was approved by the Institutional Review Board of Sichuan University (Chengdu, China). Written informed consent was obtained from both patients.

## Discussion

Ectopic pancreas, also named aberrant pancreas or heterotopia of the pancreas, is defined as pancreatic tissue lacking anatomic and vascular continuity with the main body of the pancreas. Ectopia of the pancreatic tissue is a developmental anomaly found in ~2% of all autopsies, and 70~90% of these anomalies are located in the gastrointestinal tract (10). Mediastinal localization of ectopic pancreas is extremely rare. According to previous reports (2–12) and the present cases, mediastinal ectopic pancreas can have a number of common characteristics, as follows: i) of the twelve cases, nine females and three males, all ectopias of the pancreas occurred in the anterior mediastinum; ii) the symptoms of the patients were nonspecific; iii) the masses were usually large with cystic changes due to failing to drain the exocrine fluid, inflammatory exudate and bleeding in ectopic pancreas (7); and iv) the majority of cases had a good prognosis following surgery.

As a developmental anomaly, the histogenesis of ectopic pancreas is not clear. There are two different theories on the embryogenesis of this anomalous development (10). First, ectopic pancreatic tissue in the mediastinum may be the result of the abnormal differentiation of the pluripotent epithelial cells of the ventral primary foregut, since the pancreas and the lower respiratory tract share a common embryologic origin, the primitive foregut. Second, a number of cells from the pancreatic bud may migrate and locate at a different site.

As for the symptoms, they are related to the size and location of the lesion, as well as whether an inflammation developed or not. The patient in case 1 of the present study had chest pain, cough and fever. We speculate that the symptoms resulted from the compression of the large mass and infiltrative inflammation of the ectopic pancreas, since the patient remained asymptomatic following surgery. According to the present perspective, as long as there is an ectopic pancreas present, it should be managed by surgery to prevent cystic and malignant changes, or to stop it affecting surrounding organs and tissues (7,13).

In contrast to the other cases reported, where the mass usually presents with cystic changes (2,6,7,9,10), the masses in the present study were markedly and heterogeneously enhanced, whereas necrotic and liquefied non-enhanced areas were found in the center of the mass with an irregular, well-defined margin. CT manifestations of this lesion resembled that of other neoplasms located in the anterior mediastinum, such as intrathoracic goiter, malignant thymic neoplasm and teratoma. It is difficult to differentiate between these lesions on CT. Malignant thymic neoplasm often appears as a solid mass without cystic changes and result in pleural or pericardiac effusion due to invasion of the tumor (14). Intrathoracic heterotopic thyroid gland and teratoma have similar CT features to ectopic pancreas, which appear as clear enhancement (15). Although, the goiter is derived from cervical thyroid gland, and the majority is located at a relatively high level in the anterosuperior mediastinum, and tends to extend to one side of the mediastinum (15). The anatomic association between the intrathoracic mass and the cervical thyroid gland may be confirmed through a cervicothoracic serial scan. However, teratoma is derived from pluripotent germ cells. Benign teratoma is often cystic, while malignant teratoma is usually solid. There are numerous components in a teratoma due to its various blastodermal origins, including lipids, bone tissues, teeth and hair. Therefore, a final diagnosis of teratoma can be made when these tissues are observed in the tumor (6).

In conclusion, ectopic pancreas in the mediastinum is extremely rare. When there is a mass in the anterior mediastinum with marked and heterogeneous enhancement, along with necrotic and liquefied non-enhanced areas in the center, ectopic pancreas should be considered and differentiated from other neoplasms in this region.
